# A 12-Month Follow-Up of the Effects of a Digital Diabetes Prevention Program (VP Transform for Prediabetes) on Weight and Physical Activity Among Adults With Prediabetes: Secondary Analysis

**DOI:** 10.2196/23243

**Published:** 2022-01-14

**Authors:** Ryan Batten, Meshari F Alwashmi, Gerald Mugford, Misa Nuccio, Angele Besner, Zhiwei Gao

**Affiliations:** 1 Memorial University of Newfoundland St John's, NL Canada; 2 Virgin Pulse New York, NY United States

**Keywords:** mHealth, mobile health, diabetes, DPP, diabetes prevention program, digital health, longitudinal study, prevention, weight loss, physical activity

## Abstract

**Background:**

The prevalence of diabetes is increasing rapidly. Previous research has demonstrated the efficacy of a diabetes prevention program (DPP) in lifestyle modifications that can prevent or delay the onset of type 2 diabetes among individuals at risk. Digital DPPs have the potential to use technology, in conjunction with behavior change science, to prevent prediabetes on a national and global scale.

**Objective:**

The aim of this study is to investigate the effects of a digital DPP (Virgin Pulse [VP] Transform for Prediabetes) on weight and physical activity among participants who had completed 12 months of the program.

**Methods:**

This study was a secondary analysis of retrospective data of adults with prediabetes who were enrolled in VP Transform for Prediabetes for 12 months of the program. The program incorporates interactive mobile computing, remote monitoring, an evidence-based curriculum, behavior tracking tools, health coaching, and online peer support to prevent or delay the onset of type 2 diabetes.

**Results:**

The sample (N=1095) was comprised of people with prediabetes who completed at least 9 months of the VP Transform for Prediabetes program. Participants were 67.7% (n=741) female, with a mean age of 53.6 (SD 9.75) years. After 12 months, participants decreased their weight by an average of 10.9 lbs (5.5%; *P*<.001) and increased their physical activity by 91.2 (*P*<.001) minutes.

**Conclusions:**

These results suggest that VP Transform for Prediabetes is effective at preventing type 2 diabetes through a significant reduction in body weight and increase of physical activity. Furthermore, these results suggest that the DPP remains effective 12 months after beginning the program. A prospective randomized controlled clinical study is warranted to validate these findings.

## Introduction

Diabetes is associated with considerable economic and social burden [1]. It is one of the leading causes of mortality, disability, and decreased work productivity [2,3]. The global prevalence of type 2 diabetes has been increasing in recent decades [4] as well as the rate of prediabetes [5]. In 2015, 33.9% of adults 18 years or older in the United States had prediabetes [6]. According to an American Diabetes Association panel [7,8], up to 70% of adults with prediabetes will develop type 2 diabetes.

Type 2 diabetes can be managed and prevented using lifestyle change programs. Clinical trial efficacy data demonstrated a marked reduction in progression from prediabetes to type 2 diabetes mellitus among individuals who achieved modest weight loss through lifestyle change focused on dietary change and increased physical activity [9]. Based on these findings, the Centers for Disease Control and Prevention (CDC) launched the National Diabetes Prevention Program to help individuals with prediabetes achieve 5% to 7% body weight loss [10,11]. Diabetes prevention programs (DPPs) have been widely implemented and have been shown to be effective in helping individuals reduce their weight and improve health behaviors such as engaging in physical activity and eating a balanced diet [12-16].

DPPs can reduce the risk of developing type 2 diabetes and, if scaled effectively, have the potential to reduce the prevalence of diabetes [17,18]. Barriers such as transportation and time have been associated with in-person DPPs [19]. The use of digital therapeutics for the delivery of such programs may increase program accessibility and participation [20]. DPPs have also demonstrated a return on investment by preventing diabetes and reducing the need for later stage more costly interventions [21]. Due to their scalability, digital DPPs can be a cost-effective method to lower the risk of developing type 2 diabetes.

Smartphones can deliver effective interventions among various age groups and in many disease areas, including diabetes [22-24]. Mateo et al [24] conducted a systematic review and meta-analysis to compare the efficacy of mobile phone apps with other approaches that promote weight loss and increase physical activity. The authors concluded that mobile phone app-based interventions may be useful tools for weight loss [24]. Studies have shown that innovations in health technology demonstrated positive behavior changes among patients with type 2 diabetes [25,26].

DPPs delivered via mobile health technology can result in weight loss. Chin et al [27] reported 77.9% of participants had a reduction in weight due to the use of a digital DPP, with 22.7% reducing weight by 10%. Albright and Gregg [13] demonstrated the effectiveness of a digital DPP in reducing weight by 11 pounds after 4 months of beginning the program. A systematic review examining 28 studies determined that the average weight loss was approximately 4% [28]. Weight loss in the first 6 months has been associated with a decreased risk of diabetes and associated with a decreased cardiometabolic risk and predictive [27].

**Figure 1:**
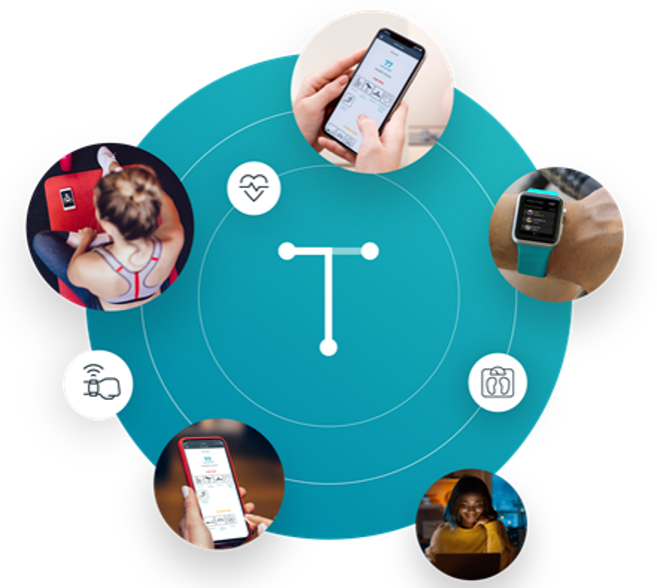
Virgin Pulse Transform for Prediabetes components: a smartphone app with digital tracking and communication tools, a wireless scale, a professional health coach, a private peer community, and an activity tracker.

Virgin Pulse (VP), a global digital health company, adapted the CDC’s Diabetes Prevention Program to a digital model to enable a highly scalable, convenient, and flexible delivery of the CDC program. VP Transform for Prediabetes integrates interactive mobile computing (ie, a smartphone app), wearable tracking devices (ie, activity tracker), remote health monitoring hardware (ie, digital scale), and professional health coaches to effectively address the complex factors that impact health behavior. The program components are described in detail later and outlined in Figure 1. Effectiveness of the digital DPP, VP Transform for Prediabetes (formerly known as Transform), was previously evaluated over a 4-month period resulting in an average weight loss of 13.3 pounds after 4 months [11].

Current research has predominantly focused on either shorter-term effects of a digital delivery model or longer-term effects of a delivery model on a small sample. This study aims to build upon the 16-week study by examining a larger sample of participants over a longer study period to assess longer-term results from program completion. Using a 12-month study period may help assess the sustainability of the early (4-month) weight loss.

## Methods

### Design and Setting

The study is a secondary analysis of data collected via the VP Transform for Prediabetes program. Deidentified data were collected from baseline to 12 months. Two outcomes were assessed: weight loss and changes in levels of physical activity. Physical activity was calculated by adding the total reported weekly minutes of physical activity (measured from the Fitbit device).

### Intervention: VP Transform for Prediabetes

VP Transform for Prediabetes is a 12-month intervention that uses the CDC’s DPP program structure by delivering the program in two phases: the 4 months of high-frequency core intervention followed by 8 months of complementary maintenance programming to support the new health behaviors.

#### Curriculum

The DPP curriculum is presented in a digital format via a smartphone app and includes survey questions, quizzes, and open-response questions. The lesson curriculum includes topics like eating balanced meals that follow the MyPlate United States Department of Agriculture [29] guidelines, benefits of physical activity and methods to increase it, stress management, social support, and how to maintain healthy lifestyle changes.

The program matches individuals with DPP-certified health coaches who motivate and guide participants to reach their health goals. Health coaches keep participant discussions on track, provide personalized feedback on food logs and physical activity progress, and conduct individualized coaching sessions using specialized techniques such as motivational interviewing through private messages, calls, and emails. Quiz responses and open responses are shared with the health coach. Lesson completion is defined as completing the quiz associated with each lesson that is delivered at the end of the content.

#### Group Support

Participants are placed into private chat groups within the smartphone app to recreate the experience of a group dynamic. An online group discussion allows participants to post questions, reply to comments, and share their experiences and progress. Group discussion is asynchronous, rather than live, to make the intervention more flexible and convenient.

#### Digital Tracking Tools

A wearable tracking device and digital scale are provided to participants. If a participant is active for more than 15 minutes, the amount of physical activity is automatically captured by the wearable tracking device. In addition, a photo-enabled food diary facilitates tracking of eating behaviors. Participants are asked to track their food by taking a picture of each meal, snack, or drink and uploading it to the app. The health coach reviews the tracking once a week and provides feedback.

### Participant Recruitment

VP Transform for Prediabetes participants were recruited via a marketing channel partner. Participants received packages in the mail that included a wireless weight scale by BodyTrace, Inc and a wearable activity tracker by Fitbit, Inc (Flex 2 model).

### Eligibility Criteria

Participants were eligible for the digital DPP if they met the following conditions: scored ≥9 on the online survey adapted from the CDC prediabetes screening tool or ≥5 on the American Diabetes Association risk screening tool and/or indicated prediabetes diagnosis through a recent blood test (self-reported, within the last 12 months); had a BMI of ≥25 kg/m^2^ (≥23 kg/m^2^ if self-identified as Asian); were ≥18 years of age; recorded their weight during the program; had a smartphone with an up-to-date operating system; had regular access to Wi-Fi; enrolled in the program between October 2017 and October 2018; had never previously participated in the program; did not have type 1 diabetes, type 2 diabetes, or end stage renal disease; and were not pregnant at the time of enrollment.

In addition to these, the following engagement criteria requirements were applied: time from the first lesson to the last lesson is at least 9 months, where a lesson is defined as completing the quiz or completing a remote session with their coach; has at least two weight readings: a baseline weight reading and a second weight reading, which takes place within a 2-week buffer from day 1 of month 12 and the last day of month 12; and engagement in the Core Phase and Maintenance Phase where engagement events include: stepping on the scale, engaging in a coaching session with a coach, posting in a group chat, logging in at least 3 meals in a lesson period, completing the quiz in 5 of the first 22 (core and biweekly) weeks. An individual had to have at least two engagement events in months 1 to 5, and at least two engagement events in 3 months between months 6 to 12.

The engagement criteria are adapted from the CDC’s Diabetes Prevention Recognition Program (DPRP) requirements (2018). According to the DPRP, participant data must meet the following criteria to be qualified for preliminary or full recognition: attend at least 3 sessions in the first 6 months and whose time from the first session to the last session is at least 9 months, and at least 60% of participants attend at least 3 sessions in months 7 to 12.

### Measures

Two outcomes were measured for this study: weight loss and physical activity. Weight loss was calculated in pounds and percent of initial body weight lost. A scale was used to measure weight, with weight loss being calculated as the weight measurement subtracted from the initial body weight. Physical activity was measured using a fitness tracker, which measured daily physical activity in minutes if the activity was at least 15 minutes long. For this study, physical activity was examined as total physical activity per week, calculated as the sum of physical activity each day for each week.

### Ethics

The Health Research Ethics Board in Newfoundland and Labrador, Canada reviewed and approved this secondary analysis.

### Statistical Analysis

#### Descriptive Statistics

Frequencies and percentages for categorical variables were used to describe participant demographics, with means and SDs for continuous variables. Analyses were conducted using SAS, version 9.4 (SAS Institute). A *P* value <.05 was considered statistically significant for all results.

#### Generalized Estimating Equations

Due to the longitudinal nature of our study design, we used a generalized linear regression with generalized estimating equations, with the exchangeable working correlation structure, to examine the significant association between time in weeks and weight and physical activity during the follow-up period. Weight loss was measured in pounds and percent of body weight lost. Data were analyzed at 6, 9, and 12 months.

## Results

### Sample Size

**Figure 2:**
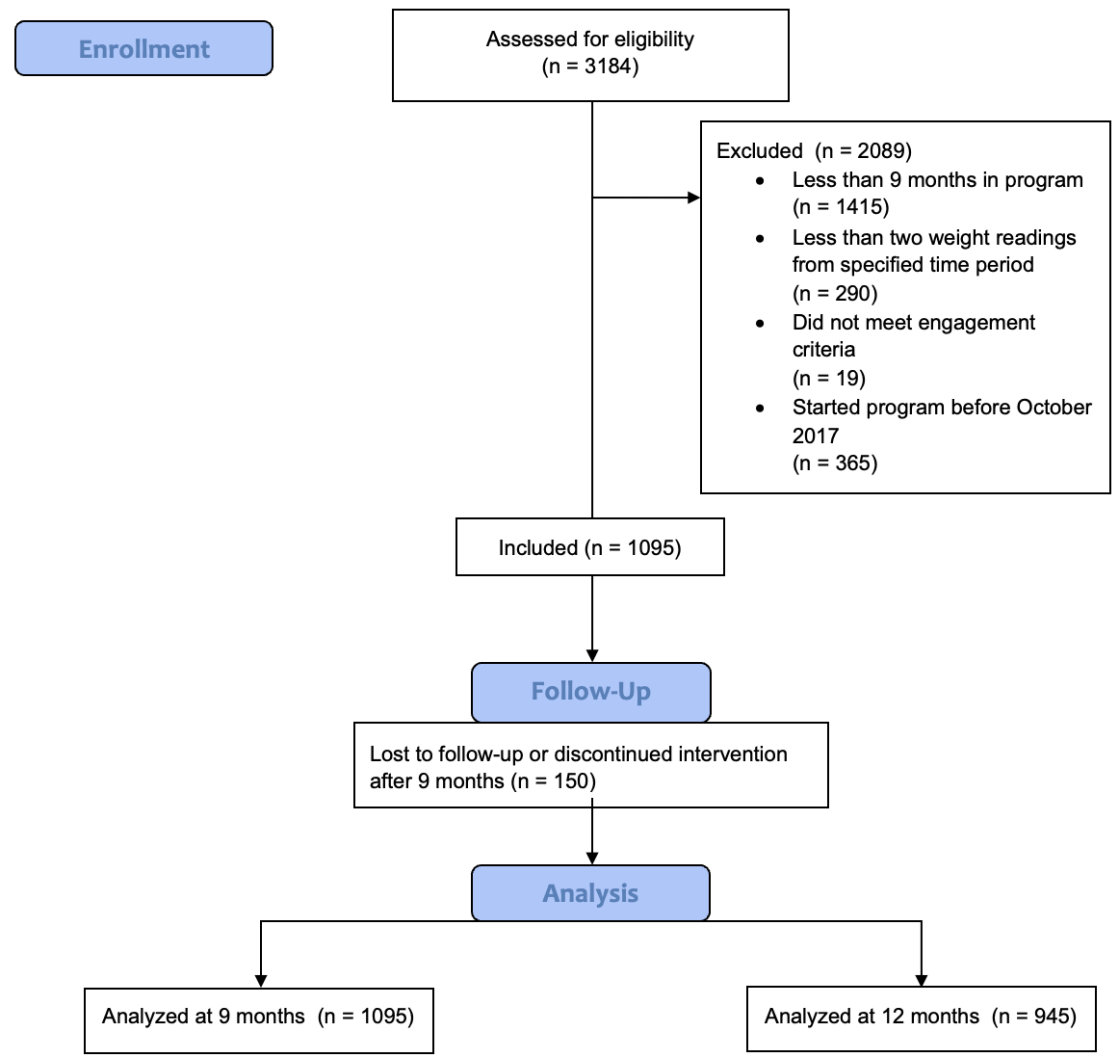
Participant flow diagram.

Of the 3184 individuals who enrolled in the Transform program, 2089 did not meet the inclusion criteria to be included in the study (Figure 2). After completing month 9 of the program, 13.7% (150/1095) of participants were lost to follow-up by month 12. This response rate is consistent with other studies using online surveys [30].

**Figure 3:**
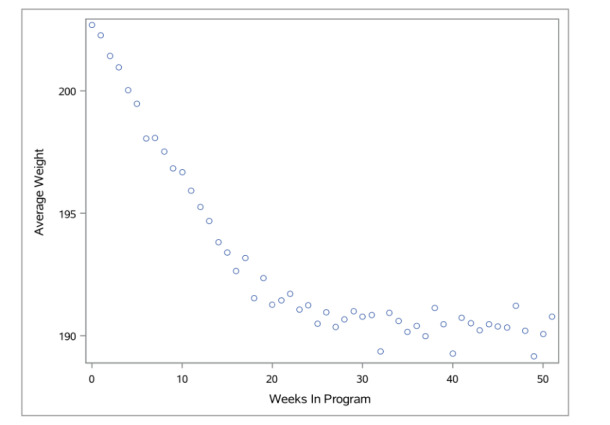
Graph of participants’ average weight.

### Demographics

A total of 1095 participants (1095/3184, 34.3%) were included in the analysis. Table 1 shows the sample demographics. Our sample had a mean age of 53.6 years and was 67.67% (n=741) female.

**Table 1:** Participant demographics.

Characteristic		Participants (n=1095)
Age (years), mean (SD)		53.6 (9.75)
Sex (female), n (%)		741 (67.67)
**Ethnicity, n (%)**		
	White	682 (62.28)
	Asian	73 (6.67)
	Black	95 (8.68)
	Other	195 (17.81)
Missing^a^, n (%)		50 (4.57)

^a^These participants had missing demographic variables.

### Weight Loss

At enrollment, the average starting weight was 204.5 (SD 42.9) lbs. After participation in the VP Transform for Prediabetes program for a minimum of 9 months, the average weight was 191.4 (SD 41.27) lbs, resulting in an average weight loss of 11.4 lbs and 5.5% weight loss. Table 2 shows the results from the generalized estimating equation. Physical activity was significantly associated with weight loss (Table 3).

**Table 2:** Weight loss and physical activity at 9 months and 12 months.

	Baseline	Mean change baseline to 9 months^a^ (n=1095)	Mean change baseline to 12 months^a^ (n=945)
Weight (lbs), mean (SD)	204.49 (42.92)	–13.30 (0.56)	–10.88 (0.62)
Weight loss (%), mean (SD)	N/A^b^	–6.60 (0.28)	–5.47 (0.31)
Participants with ≥5% weight loss, n (%)	N/A	639 (58.35)	552 (58.41)
Weekly physical activity (minutes)	66.97 (3.84)	116.45 (11.47)	91.22 (12.36)

^a^Adjusted mean and SE from generalized estimating equation models.

^b^N/A: not applicable.

**Table 3:** Results from generalized estimating equation.

Outcome measure and parameter		β (SE)	95% CI	*P* value
**Weight loss (lbs)**				
	Intercept	–11.44 (0.29)	–12.02 to –10.87	<.001
**Weight (lbs)**				
	Intercept	199.88 (1.28)	197.38 to 202.39	<.001
	Weeks	–0.23 (0.012)	-0.2565 to –0.2085	<.001
**Weight loss (%)**				
	Intercept	–5.47 (0.13)	–5.71 to –5.22	<.001
**Weight loss (%)**				
	Intercept	–3.14 (0.089)	–3.26 to –2.91	<.001
	Weeks	–0.11 (0.0054)	–0.1202 to –0.0989	<.001
**Physical activity^a^** **(minutes)**				
	Intercept	132.94 (4.08)	124.94 to 140.95	<.001
**Physical activity^a^** **(minutes)**				
	Intercept	167.80 (5.32)	157.38 to 178.23	<.001
	Weeks	–1.64 (0.13)	–1.90 to –1.38	<.001
**Weight (lbs)**				
	Intercept	200.43 (1.28)	197.91 to 202.94	<.001
	Weeks	–0.24 (0.013)	–0.26 to –0.21	<.001
	Physical activity^a^	–0.0032 (0.0010)	–0.0052 to –0.0013	.001
**Weight loss (%)**				
	Intercept	–2.87 (0.12)	–3.05 to –2.59	<.001
	Weeks	–0.11 (0.0056)	–0.1230 to –0.1012	<.001
	Physical activity	–0.0016 (0.0005)	–0.0025 to –0.0007	.001

^a^Physical activity measured as total weekly physical activity in minutes.

At baseline (n=1095), participant’s average total physical activity per week was 66.97 minutes. After participating in the 16-week core curriculum of the program, the average increased to 154.90 minutes per week (n=1095). At the end of the study period, the average number of physical activity minutes per week was 132.94 (n=945; Table 4).

**Table 4:** Physical activity results.

Outcome measure and parameter				β (SE)		95% CI		*P* value
**Baseline**								
	**Physical activity**							
		Intercept	66.97 (3.84)		59.45 to 74.50		<.001	
**End of 16-week core curriculum**								
	**Physical activity^a^ (minutes)**							
		Intercept	154.90 (4.80)		145.49 to 164.31		<.001	
	**Physical activity^a^ (minutes)**							
		Intercept	126.11 (5.50)		115.32 to 136.90		<.001	
		Weeks	3.76 (0.68)		2.43 to 5.10		<.001	
**After 6 months (24 weeks)**								
	**Physical activity^a^ (minutes)**							
		Intercept	149.97 (4.54)		141.06 to 158.87		<.001	
	**Physical activity^a^ (minutes)**							
		Intercept	148.30 (4.99)		138.53 to 158.07		<.001	
		Weeks	0.15 (0.27)		–0.38 to 0.6852		.57	
**End point**								
	**Physical activity^a^ (minutes)**							
		Intercept	132.94 (4.08)		124.94 to 140.95		<.001	

^a^Physical activity measured as total weekly physical activity in minutes.

**Figure 4:**
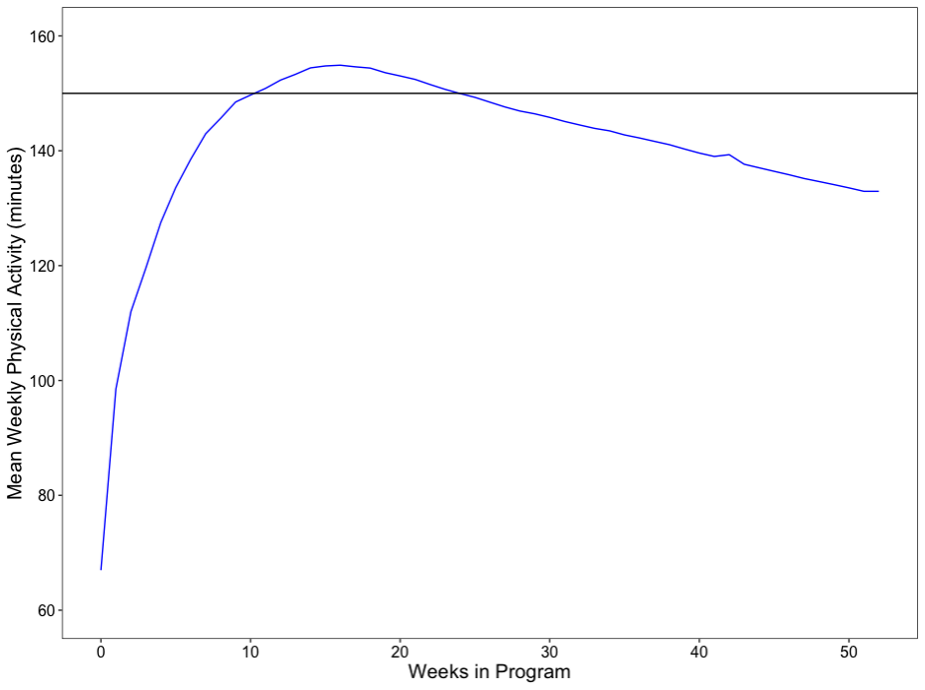
Graphical representation of physical activity.

At baseline, the mean physical activity was 66.9 minutes per week (n=1095). After completing the first 16 weeks of the program, participant’s average physical activity per week exceeded the 150 minutes per week goal at 154.9 minutes per week (n=1095). At the end of the study period, the physical activity decreased to 132.94 minutes (n=945); however, participants improved their physical activity by 88 minutes per week compared to baseline after completing the program. Mean weekly physical activity is shown in Figure 4, with participants meeting or exceeding 150 minutes after completing the 16-week core curriculum then decreased during the maintenance phase of the program.

## Discussion

### Principal Findings

Overall, participants either attained or surpassed the program goals. Weight and physical activity improved after completing 12 months of the VP Transform program. Mean weight decreased throughout the program (Figure 3). Similarly physical activity increased after participating in the 16-week core curriculum, with a slight decrease between month 4 and 12 after completing the program (Figure 4). After 12 months of completing the program, mean weight declined by 10.9 lbs (5.47% of total body weight, n=945), while physical activity per week increased by 65.97 minutes (n=945) compared to baseline. This illustrates that VP Transform for Prediabetes is effective at reducing body weight and improving physical activity after completing the program.

### Comparison With Previous Work

Prior data demonstrated the effectiveness of VP Transform after 4 months of completing the program [11]. We extend these findings by reporting results 12 months after beginning the program among a larger cohort of participants (n=945) compared to the previously reported study (n=273).

The mean weight declined by 10.9 lbs (5.47% of total body weight) among VP Transform for Prediabetes participants (n=945) who completed 12 months of the program. These are similar to, but slightly higher than, results reported in some previous studies. Sepah et al [31] reported a weight loss of 10 lbs after 12 months or 4.7% weight loss (n=187), Moin et al [30] found a mean weight change of 8.8 lbs after 12 months or 3.7% weight loss (n=268), and Gilis-Januszewska et al [32] found an average weight loss of 4.9 lbs at the 12-month follow-up among 105 participants (average percentage of weight loss not reported).

A systematic review of 22 studies analyzing diabetes prevention lifestyle interventions concluded an average mean weight loss of 5.1 lbs after 12 months [33]. Clinically significant weight loss is defined as at least a 5% reduction in weight from baseline levels [34] and is associated with improvements in cardiometabolic risk factors, such as reduction in blood lipids and improved insulin response [35-37]. Our results suggest that VP Transform for Prediabetes is effective at reducing participants’ risk of developing type 2 diabetes through sustained and clinically meaningful weight loss from baseline to 12 months.

It is difficult to compare the results of this study with previously published literature due to different interventions and duration examined. A systematic review by Cotterez et al [37] reported that only 1 study found statistically significant differences in activity levels for participants in web-based programs compared to those in a non–web-based control group. Furthermore, there is limited objective data regarding the effect of digital DPPs on physical activity [37].

### Limitations and Strengths

This study was a retrospective longitudinal cohort study. As a result, results from this study may be due to factors other than VP Transform for Prediabetes. Participants were predominantly female, which may affect the generalizability of the study to both sexes. Additionally, there was also no control group, which would minimize the effect of all confounding variables and would strengthen the correlation between the intervention and the outcomes.

Although physical activity was measured, the intensity level was not differentiated between moderate and vigorous. This affected the ability to evaluate VP Transform for Prediabetes against the physical activity goal of 150 minutes of moderate physical activity each week. Using a Fitbit for physical activity does not account for when an individual is not wearing it. As a result, a reading of 0 may respond to an individual not wearing the device.

The primary strength of this study was the use of objective measurements from activity trackers and a weight scale. This study had a large sample size, which increases the generalizability of the results. Additionally, the relative lack of attrition during the 12-month period indicates that the impact found in this study is likely sustainable over time, which is a key feature of success in impacting chronic conditions such as diabetes.

### Future Studies

Future studies should examine other aspects of the digital DPP, such as work productivity metrics, sleep, and diet. An experimental study should be included to assess the impact of VP Transform for Prediabetes factors on additional health risk outcomes and potential confounding variables such as ethnicity, income, geography, and gender. Examining the effects of specific engagement data could also be included. Lastly, a study examining the economic impact of VP Transform for Prediabetes would be beneficial.

### Conclusion

VP Transform for Prediabetes significantly reduces body weight and results in an increase in total weekly physical activity minutes. The study’s findings highlight the effectiveness of the program in promoting meaningful changes to participants’ behaviors, leading to a reduction in their risk for type 2 diabetes.

## Abbreviations

CDC: Centers for Disease Control and Prevention
DPP: diabetes prevention program
DPRP: Diabetes Prevention Recognition Program
VP: Virgin Pulse
